# Gracilis flap and partial colpocleisis of Kahr for pelvic organ prolapse after anterior exenteration: A case report

**DOI:** 10.1016/j.crwh.2024.e00673

**Published:** 2024-11-28

**Authors:** Greta Lisa Carlin, Sören Lange, Werner Haslik, Harun Fajkovic, Engelbert Hanzal

**Affiliations:** a Department of General Gynecology and Gynecologic Oncology, Medical University of Vienna, Austria; b Department of Gynecology, University Hospital Zurich, University of Zurich, Zurich, Switzerland; c Department of Urology, Medical University of Vienna, Austria; d Department of Urology, University Hospital St. Pölten, Austria; e Medical University of Zurich, Switzerland

**Keywords:** Anterior exenteration, Colpocleisis, Gracilis flap, Pelvic exenteration, Pelvic floor reconstruction, Radical cystectomy complications

## Abstract

Anterior exenteration is a radical surgical option for treating locally advanced pelvic malignancies when alternative treatments are deemed ineffective or inappropriate. Due to its nature as an ablative treatment, interference with supportive structures of the pelvic floor can result in pelvic organ prolapse.

A 70-year-old woman presented with prolapse after radical cystectomy and following two unsuccessful attempts at Le Fort colpocleisis, the second of which was further complicated by rupture of the vaginal cuff. After exploratory laparotomy to evaluate pelvic adhesions and potential tumor recurrence, the necrotic vaginal apex was excised via the vaginal route, and a musculus gracilis flap was created to cover the levator hiatus in a *Z*-shaped pattern. The introitus was then narrowed by partial colpocleisis of Kahr. The postoperative course was uneventful and high patient satisfaction and an adequate anatomic result were found at one-year follow-up.

There is a scarcity of literature regarding the optimal treatment for pelvic organ prolapse after anterior exenteration, and to our knowledge this is the first published report of the use of a gracilis flap combined with partial colpocleisis of Kahr with a satisfactory outcome in this complicated situation. This case underscores the importance of a multidisciplinary approach in managing prolapse after radical cystectomy, showcasing the successful integration of expertise across gynecology, urology, and reconstructive surgery.

## Highlights

• Management of recurring prolapse after radical cystectomy in a 70-year old woman is described.
• Two earlier repairs had failed, one causing rupture of the vaginal cuff.
• Management involved a combination of Gracilis flap and Kahr colpocleisis.
• There was no recurrence over one-year follow-up.
• The case underscores the importance of a multidisciplinary approach.

## 1 Introduction

Pelvic exenteration (PE) was first introduced as a palliative procedure for advanced pelvic malignancies and later became a radical surgical option when alternative treatments are inadequate [1]. Radical cystectomy (RC) is often performed for muscle-invasive bladder carcinoma but it can disrupt the pelvic floor support structures and lead to complications such as pelvic organ prolapse (POP) [2].

Managing POP post-RC is particularly challenging due to altered pelvic anatomy, bowel adhesions, and the presence of urinary diversions such as ileal conduits [3]. Conservative management is often not feasible due to frequent pessary dislocation or pain. On the other hand, the optimal surgical management of POP in this patient population remains unclear. There have been only a few small case series published on surgical repair for POP in this specific population, with a focus on transvaginal approaches.

This case report demonstrates the complexities of managing POP post-RC and highlights the benefits of a multidisciplinary approach. We present a case of native tissue repair of a vaginal prolapse after anterior exenteration with two failed previous attempts at Le Fort colpocleisis [4,5].

## 2 Case Presentation

A 70-year-old woman was referred with recurring POP after robotically assisted anterior exenteration with an ileal conduit for locally advanced bladder cancer at 66 years of age and two failed attempts at Le Fort colpocleisis one and two years later; the latter was further complicated by cuff rupture and bowel herniation. During clinical examination, total POP with thin atrophic and partially necrotic ulcerated vaginal skin gave POP-Q scores [6] of Aa +3, Ba +6, C + 6, gh 5, pb 3, tvl 10, Ap −1, and Bp −1, while the levator ani was intact on both sides (Oxford muscle strength grading 3/5). Biopsies of suspicious areas of the vaginal wall were performed and revealed tumor-free, nonspecific ulcers and granulation tissue. Pessary treatment was deemed unfeasible due to poor tissue quality. The case was reviewed by a multidisciplinary pelvic floor board, including urogynecologists, urologists and plastic surgeons. The consensus was to proceed with operative closure of the pelvic floor defect using either a rectus abdominis or gracilis flap, depending on intraoperative findings [7,8].

**Fig. 1:**
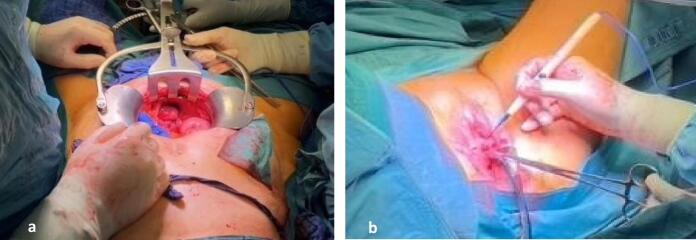
a) Median re-re-laparatomy: few adhesions, no tumor reoccurrence, b) Opening and resection of anterior vaginal wall.

**Fig. 2:**
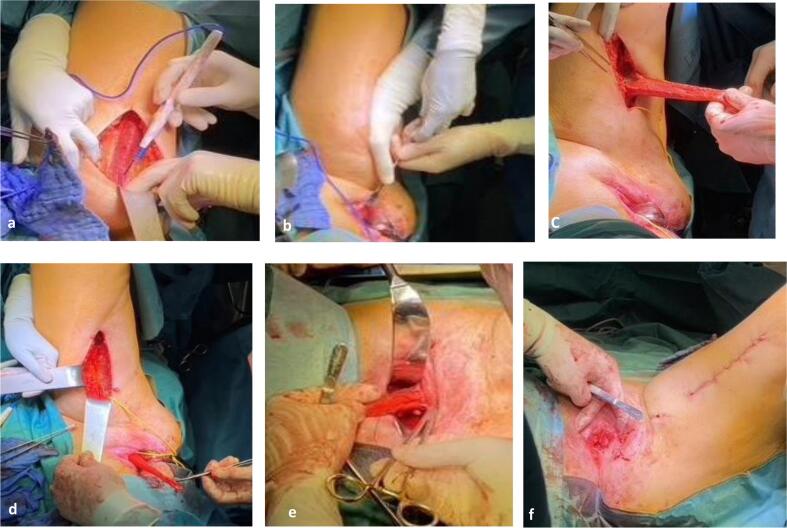
a) Locating *M. gracilis*, b) Tunnelling behind the external genital muscles, c) Mobilisation of gracilis flap, d) Rotation of the gracilis flap through the tunnel behind the external genital muscles, e) S-shaped coverage of the urogenital hiatus, f) Partial colpocleisis of Kahr.

**Fig. 3:**
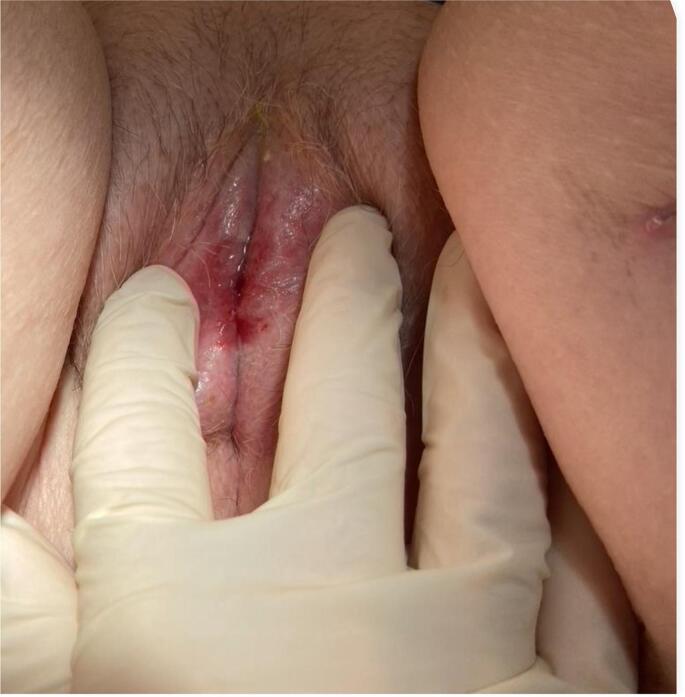
Introitus at 1-month follow-up.

**Fig. 4:**
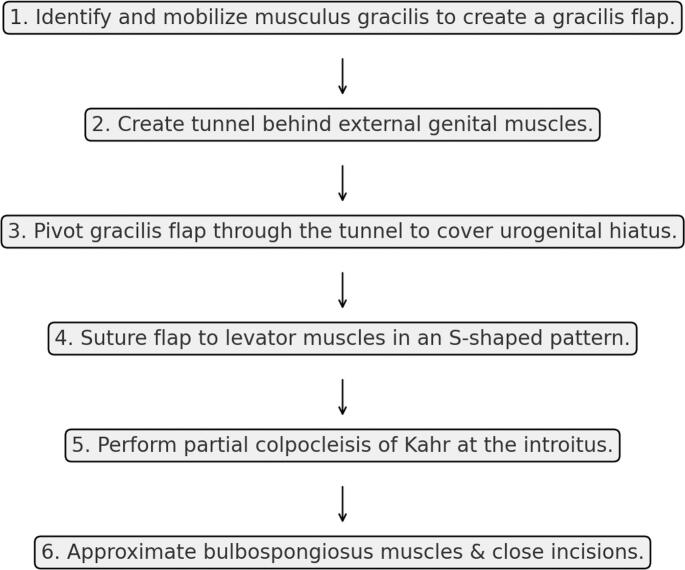
Diagram of the steps for Gracilis flap and partial colpocleisis of Kahr for pelvic organ prolapse after anterior exenteration.

After discussion with the patient and obtaining informed consent, surgery was performed by a multidisciplinary team, consisting of two urogynecologists, a urologist, and a plastic surgeon. An exploratory median re-re-laparotomy revealed few pelvic adhesions, a partially necrotic vaginal cuff and a well-functioning and otherwise unremarkable ileum conduit. Any tumor recurrence was effectively ruled out. After carefully mobilizing two small bowel loops that were attached to the vaginal wall, the greater omentum was mobilized and brought into the small pelvis to optimize blood perfusion, and the decision to proceed via the vaginal route was made (Fig. 1a). The vaginal cuff was opened from below, and as little tissue that was sufficiently vascularized was found, subtotal resection of the vagina was carried out (Fig. 1b). Then the gracilis muscle on the inside of the left thigh was located (Fig. 2a) and a tunnel behind the external genital muscles was created (Fig. 2b). After separating the left gracilis muscle from its nerve supply and rechecking the vascular situation, the flap was mobilized and pivoted through the tunnel toward the genital hiatus (Figs. 2c & 2d). Subsequently, the gracilis flap was used to cover the defect of the urogenital hiatus by attaching it through several single polyglactin 2.0 sutures in an S-shaped fashion to the inside of the levator muscle (Fig. 2e). The remaining vaginal wall and incision on the left thigh were then closed continuously with polyglactin 2.0. Finally, partial colpocleisis of Kahr was performed via a U-shaped deep incision at the introitus from 3 to 9 o'clock to expose the bulbospongiosus muscles on both sides [9] (Fig. 2f). These were approximated in two layers with polyglactin 2.0 in a continuous fashion with subcuticular skin closure, leaving a residual introitus, which was just large enough to be passed by an index finger and thus led to a high and strong perineum (Fig. 3a). All steps are summarized in Fig. 4.

The patient remained for observation over one night in the intensive care unit. Her postoperative course was uneventful, and six days after the procedure she was discharged from the hospital. Good patient satisfaction, wound healing and anatomic results were found at one- and three-month follow-up. During an phone follow-up after nine months and after one year, the patient expressed general happiness with the results of her surgery, no symptoms of POP recurrence and the absence of pain.

## 3 Discussion

The exact prevalence of POP in women who have undergone radical cystectomy (RC) is unknown. Despite the limited literature on POP repair in women with previous RC, it is evident that conventional approaches to managing POP become more challenging in this group of patients. Conservative management with a pessary might typically provide symptomatic relief; however, in post-RC women, the use of a pessary can be difficult or even impossible due to the shortened anterior vaginal wall and decreased vaginal capacity. This was also the case for this patient. As she had already undergone two previous attempts at colpocleisis in the two years after her RC for advanced bladder cancer, conservative treatment was not feasible. As the decision to proceed with surgery was made, further preoperative diagnostics, i.e., imaging, were deemed unnecessary since surgical exploration would have to take place to evaluate the vitality and blood perfusion of the local tissue.

The management of POP after RC involves complex decision-making, requiring input from various specialties. Urogynecologists contribute expertise in pelvic floor anatomy and repair techniques, while urologists address the challenges posed by urinary diversions and altered pelvic anatomy. The involvement of a plastic surgeon is crucial for reconstructive options, such as flaps, which may be necessary when native tissue repair is inadequate.

There is a scarcity of literature regarding the optimal surgical treatment for POP after anterior exenteration, with most of the literature comprising individual case reports. Treatment options vary from native tissue repair to reconstruction with mesh to obliterative procedures. However, the use of synthetic mesh may not be suitable where there is radiogenic alteration or otherwise poor vascularity of tissue. Further, it is important to note that mesh-related complications can occur, such as protrusion or erosion through the vagina or bowel with severe consequences.

Standardised reconstructive native tissue repairs seemed unfeasible, and even obliterative procedures had failed twice in this patient. The possibility of creating a neovagina versus a colpocleisis was also discussed with the patient, but since she reported no desire for penetrative sexual activity, she decided in favor of an obliterative procedure.

The plastic surgeon of the multidisciplinary team recommended a rectus abdominis or a gracilis flap as the most feasible options. The advantages and disadvantages of each flap technique depend mainly on the local anatomical conditions.

The vertical rectus abdominis myocutaneous (VRAM) flap is a well-known method used to reconstruct pelvic and perineal defects that cannot be closed directly [10,11]. The VRAM flap benefits from a dual blood supply from either the superior or inferior epigastric arteries, allowing it to be moved in either a cranial or caudal direction. Additionally, due to their larger muscle bulk, VRAM flaps are more effective at filling larger areas of empty space in the pelvic region than other muscles [7,12]. Another option for perineal reconstruction is the use of flaps originating from the thigh. However, the anterolateral thigh flap and posterior fasciocutaneous thigh flap may not provide enough tissue to repair extensive perineal defects with empty spaces. On the other hand, the gracilis flap is a well-documented substitute for the VRAM flap in genital and perineal reconstruction [8]. The gracilis flap offers the advantage of minimal complications at the donor site and can be easily elevated during the procedure.

The patient was also informed in detail that an exact decision as to which type of flap (rectus flap versus gracilis flap) was the best option in her case could be made only intraoperatively. The patient agreed to both options. During explorative laparotomy, the vascular situation of the abdomen (musculus rectus) and vaginal wall perfusion were assessed and deemed insufficient for the rectus flap. Thus, the decision to proceed with a gracilis flap was made. In addition, a colpocleisis after Kahr was performed. In 1937 Kahr described a simple technique to significantly narrow the introitus: by dissecting down to the external genital muscles and perineum at the level of the hymen from 11 to 1 o'clock and bringing the structures together in the midline, leaving only a small opening for the external urethral meatus [12]. This procedure can be performed under local anesthetic within minutes and is thus ideal for a frail, elderly population. A slight modification of this technique with less radical introital narrowing to complement LeFort's colpocleisis was adopted and used to close up the space below the gracilis flap.

Treating POP in post-RC women is challenging. However, urologists can lower the incidence of this complication by gaining a better understanding of the supportive structures of the female genital tract, minimizing the excision of important ligaments and endopelvic fasciae during surgery, and, if necessary, repairing them at the same time, i.e., by reattaching the uterosacral/lateral cervical ligamentous complex to the vaginal vault to prevent vaginal vault prolapse in the future [13].

In addition, providers caring for post-RC women should also know the factors that can increase the risk of developing POP or perineal herniation. Pre-surgery risk factors include gravity, parity, bulge symptoms, history of hernia, previous prolapse surgery, hysterectomy, and obesity [[14], [15], [16], [17]]. Post-surgery factors include small bowel obstruction or ileus, chronic constipation or cough, wound opening, weight gain, and overall physical weakness [2]. By improving the identification of this complication, the quality of life of women suffering from it can be significantly increased.

The complexity of managing POP in post-RC patients lies in the altered pelvic anatomy and the need for a tailored approach. This case demonstrates the successful integration of a multidisciplinary team in addressing these challenges. The positive outcome achieved in this case supports the use of gracilis flap combined with partial Kahr colpocleisis as a viable option for managing complex POP cases.

## 4 Conclusion

To our knowledge, this is the first reported case of a gracilis flap with partial Kahr colpocleisis for total POP after anterior exenteration. It highlights the importance of a multidisciplinary approach in managing POP following anterior exenteration. Furthermore, it shows that it is possible to perform vaginal native tissue pelvic reconstruction in patients who have undergone pelvic exenteration and multiple previous POP repairs. The successful outcome achieved with a gracilis flap and partial Kahr colpocleisis underscores the value of collaboration between urogynecologists, urologists, and plastic surgeons. Such an approach ensures comprehensive care, tailored to the unique anatomical and functional challenges posed by post-RC prolapse, ultimately improving patient outcomes.
